# Special Issue “Bacterial Toxins and Cancer”

**DOI:** 10.3390/ijms25042128

**Published:** 2024-02-09

**Authors:** Sara Travaglione, Francesca Carlini, Zaira Maroccia, Alessia Fabbri

**Affiliations:** Department of Cardiovascular, Endocrine-Metabolic Diseases and Aging, Istituto Superiore di Sanità, 00161 Rome, Italy; sara.travaglione@iss.it (S.T.); francesca.carlini@iss.it (F.C.); zaira.maroccia@iss.it (Z.M.)

Infection is a major contributor to the development of cancer, with more than 15% of new cancer diagnoses estimated to be caused by infection [1,2,3]. Eleven infectious biological agents are classified as “Group 1 carcinogens” in the Monographs of the International Agency for Research on Cancer. These include viruses, such as Epstein–Barr, hepatitis B and C viruses, human immunodeficiency virus type 1, the *Helicobacter pylori* bacterium, and helminths infections [4]. Bacteria are increasingly recognized in the field of oncology, and several studies have been conducted on the pro-carcinogenic potential of chronic bacterial infections in certain districts [5,6,7,8,9,10]. Virulence factors produced by pathogenic bacteria as protein toxins favor colonization and diffusion in the host, disturbing the physiological balance and causing a state of disease. In this context, some bacterial toxins have been studied in recent years because of their association with cancer initiation and progression [11,12,13,14,15,16,17,18]. These toxins generally induce changes in cells that are reminiscent of cancer cells and possess highly specific enzymatic activity on key molecular targets. In the rapidly evolving landscape of molecular biology studies, bacterial toxins are also being considered as novel potential therapeutics, particularly in the field of anti-cancer therapies [19,20,21,22,23].

The current Special Issue, entitled “Bacterial Toxins and Cancer”, collects insightful reviews and research articles on the association between bacterial toxins and cancer initiation and progression, as well as the ability of some bacterial toxins to serve as useful tools for molecular biology studies by manipulating key host cellular processes. Six recent research papers on different topics have been included, specifically three research articles [24,25,26] and three reviews [27,28,29], with over 30 different contributors, that explore recent advances in the field of bacterial toxins and cancer in both the aforementioned areas. In fact, four papers address the potential anti-cancer activity of toxins [24,25,26,27], and two focus on the possible involvement of these virulence factors in cancer initiation and progression [28,29].

This summary provides a concise overview of the valuable papers in this Special Issue, with the goal of increasing their impact and citation rates.

In the field of toxins with anti-cancer potential, Xu et al. [24] focus on two interesting therapeutic targets that are overexpressed in pancreatic cancer (PC) and associated with a poor prognosis: the human epidermal growth factor receptor 3 (HER3) and the epithelial cell adhesion molecule (EpCAM). The authors analyze the anti-tumor effect of Ec1-LoPE (a fusion protein consisting of the EpCAM targeting agent Ec1 fused to the deimmunized cytotoxic agent LoPE toxin from *Pseudomonas aeruginosa*) and MM-121 (Seribantumab, an antagonist that blocks the ligand-dependent activation of HER3), either as monotherapy or in combination, in an in vivo mouse model of PC. The hypothesis is that, by targeting two different molecular mechanisms with a combined treatment, the cytotoxic effect on cancer cells would be enhanced, off-target toxicities would be potentially reduced, and the development of drug resistance would be prevented. The results show a significant effect of the combined therapy on the outcome parameters, but not on the survival curve of the treated mice. The authors conclude that the combined therapy with Ec1-LoPE and MM-21 is feasible for the treatment of PC, one of the most aggressive tumors with increasing incidence and a high mortality rate [30,31]. However, further investigation and optimization of the pharmacokinetic parameters are required. 

The paper by Jun et al. [25] addresses one of the major limitations of the immunotoxins (ITs) in cancer therapy, namely, the concomitant expression of tumor antigen target receptors on normal tissues, resulting in on-target/off-tumor toxicity. To increase the selectivity of ITs for tumors over normal tissues, the authors propose a strategy to customize the anti-EGFR affinity of a monobody-based IT bearing a fragment of *P. aeruginosa* exotoxin A (PE24), termed ER-PE24, to distinguish solid tumors, which commonly overexpress EGFR, from normal tissues.

The authors compare the anti-tumor efficacy and toxicity of a panel of EGFR-targeting monobody-based PE24 toxins with different EGFR affinities. They found that ER/21-PE24, which is an intermediate EGFR affinity, shows strong anti-tumor activity with low systemic toxicity. In vitro and in vivo, they demonstrate eight- and fourfold improved therapeutic indices, respectively, compared to ER/0.2-PE24, which has the highest EGFR affinity.

The authors emphasize the importance of adjusting the EGFR affinity of EGFR-targeted ITs based on EGFR expression levels on tumors to maximize the therapeutic window while minimizing the on-target/off-tumor adverse effects.

Codolo et al. [26] present their findings regarding melanoma reduction after a treatment based on a protein toxin from *Helicobacter pylori* in an in vivo model.

The *H. pylori* neutrophil-activating protein (HP-NAP) has immunomodulatory properties [32] and may have potential therapeutic applications in the treatment of metastatic breast cancer and neuroendocrine tumors. It has been shown that HP-NAP inhibits the growth of bladder cancer and enhances the anti-tumor activity of oncolytic viruses by activating cytotoxic Th1 responses [33,34,35].

The authors investigate the potential for HP-NAP to exert its anti-tumor effect by modulating the activity of innate immune cells. Analyzing the larval stage of zebrafish, before the maturation of adaptive immunity, the authors demonstrate that HP-NAP administration effectively inhibits tumor growth and metastasis, increasing overall animal survival. This effect is accompanied by a significant recruitment of macrophages with pro-inflammatory profiles at the tumor site.

Therefore, this research indicates that reprogramming tumor macrophages could be an innovative strategy to successfully counteract melanoma growth, even in the absence of the acquired/specific branch of the immune system. Additionally, the bacterial protein HP-NAP could become a new biological therapeutic agent for the treatment of metastatic melanomas [36,37], either alone or in combination with immune checkpoint blockers.

Marquez-Lopez and Fanarraga [27] propose a review article on the potential uses of bacterial AB toxins in cancer therapy [38]. The authors describe the structure and mechanisms of action of the diphtheria (Dtx) [39], anthrax (Atx) [40], shiga (Stx) [41], and cholera (Ctx) [42] toxins and discuss the potential development of innovative and precise applications in oncology based on engineered recombinant AB toxins.

AB toxins are toxic molecules that strongly bind to cell receptors, some of which are overexpressed by both tumor cells and neovascular endothelial cells in solid tumors. Due to their modular structure, AB toxins can be genetically engineered to develop high-affinity compounds that can recognize target cells and facilitate the translocation of various therapeutic payloads into the cell. Among the toxins being considered, Stx is widely used both as an imaging and contrast agent, and it is also used as an attractive antineoplastic targeting tool [43]. Atx is the second most utilized, while the applications of Dtx and Ctx in cancer therapy are not as advanced as those of other toxins. Although there are several critical drawbacks that need to be addressed before the widespread use of engineered recombinant AB toxins in oncology, the results reported in this review suggest the possibility of developing innovative and promising applications in cancer treatment.

Markelova et al. [28] review the potential malignant role of toxins in the onset and progression of carcinogenesis, with a focus on cyclomodulins and bacterial metabolites as important factors in bacterial survival and interactions with the host organism. Strong associations between bacterial pathogens and cancer incidence are well known. Many bacterial factors have been identified that mediate the malignant transformation of host cells, including toxins, cell surface components, and effector proteins [44]. These factors are capable of causing DNA damage, increasing genome instability, and interfering with cancer-associated regulatory pathways. Even commensal bacteria, which have co-evolved with their hosts and produce a number of compounds that support various physiological processes in the human body, are capable of overcoming protective host responses and exerting pathological effects when external and/or internal factors intervene to disrupt macroorganism–metabolite homeostasis [45]. In this way, they influence the onset or development of pathological conditions, including neoplasia.

The authors emphasize the role of cyclomodulins and other bacterial metabolites in the development of pathological effects, including the onset and progression of malignant transformation in the host organism.

Finally, the review proposed by Fettucciari et al. [29] focuses on the senescence induced by *Clostridium difficile* toxin B [46] as a pathological player in the onset of colorectal cancer (CRC). The B toxin is responsible for the major pathogenic effects of *C. difficile* infection (CDI) [47], and is associated with irritable bowel syndrome (IBS) and inflammatory bowel diseases (IBDs) [48]. In addition, the B toxin has the ability to induce and increase the burden of senescent cells [46,49]. The authors discuss how the induction and accumulation of senescent cells in IBS and/or IBD after CDI and its recurrences could be a potential factor in the development of CRC. The initial accumulation of senescent cells may exert an anti-tumor effect by inducing the clearance of pre-malignant cells by immune cells recruited by some components of the senescence-associated secretory phenotype (SASP). However, in conditions where senescent cells accumulate (such as chronic injury, deregulation of immune surveillance, aging, etc.), the same SASP factors may promote tumor growth and drive cells towards complete CRC transformation [50,51]. Finally, the authors propose the elimination of senescent cells and/or neutralization of SASP as a possible therapeutic approach for CRC.

In summary, the articles included in this collection cover important research areas in the field of bacterial toxins and cancer. The field of research is still in its infancy, but bacterial toxins have proven to be highly versatile, acting both as carcinogens and, when appropriately modified, as highly specific anti-cancer agents.
